# Reperfusion hemorrhage following PCI – quantification with T2* imaging and impact on area at risk assessment

**DOI:** 10.1186/1532-429X-11-S1-O32

**Published:** 2009-01-28

**Authors:** Declan P O'Regan, Rizwan Ahmed, Clare Neuwirth, Yvonne Tan, Giuliana Durighel, Jo V Hajnal, Imad Nadra, Simon J Corbett, Stuart A Cook

**Affiliations:** 1 Imperial College, London, UK; 2 Imperial College Healthcare NHS Trust, London, UK; 3 Southampton University Hospitals NHS Trust, Southampton, UK

**Keywords:** Percutaneous Primary Coronary Intervention, Microvascular Obstruction, Late Enhancement, Myocardial Edema, Myocardial Salvage

## Introduction

Occlusion of a coronary artery leads to myocardial tissue edema in the vascular bed downstream of the vessel. The extent of hyperintense edema on T2-weighted images allows the area at risk (AAR) from ischemic injury to be retrospectively determined. However, reperfusion of severely ischemic myocardium also leads to interstitial hemorrhage and this may be an important marker for irreversible microvascular damage.

## Purpose

We assessed the feasibility of using T2* mapping to quantify regions of myocardial hemorrhage following percutaneous primary coronary intervention (PPCI) for acute myocardial infarction. We also hypothesized that myocardial hemorrhage would lead to an underestimate of the AAR on T2-weighted imaging using conventional signal threshold criteria.

## Methods

Fifteen patients who had recently undergone PPCI within the previous 7 days were imaged. Left ventricular function was assessed with conventional cine sequences. Myocardial edema was imaged with a T2-weighted STIR sequence. Myocardial haemorrhage was imaged with a black-blood multiecho T2* sequence using navigator respiratory-gating. Microvascular obstruction (MVO) and late enhancement were imaged at 1 minute and 15 minute delays respectively using a 3 dimensional inversion-recovery sequence.

The area of myocardial edema on the T2 STIR images was measured with a boundary detection tool. This was compared to a conventional signal intensity threshold method using 2, 3 and 5 standard deviations (sd) above the mean of remote normal myocardium. A salvage index was calculated as the proportion of the AAR that did not show late enhancement. T2*-mapping of the left ventricle was performed using a threshold of 20 ms to define the presence of hemorrhage.

## Results

**Figure 1:**
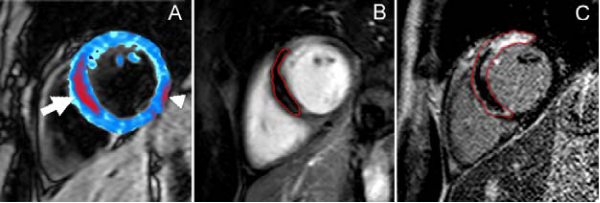
(A) T2* map acquired 2 days post-PCI. Pixels with a T2* <20 ms are shown in red and demonstrate the region of post-reperfusion hemorrhage (arrow). Susceptibility artifact (arrowhead). (B) The hemorrhage corresponds to the area of MVO (red contour) shown on the early enhancement image. (C) Myocardial necrosis (red contour), and residual MVO (black core), is shown on the late enhancement image

**Figure 2:**
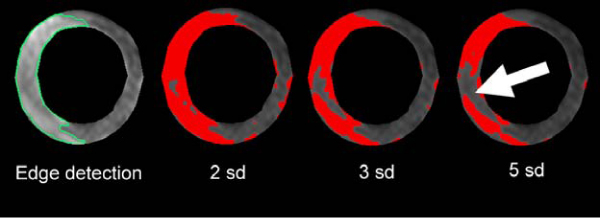
**T2-STIR images in the same patient as in Figure 1**. Boundary detection identifies the myocardial edema (green line) which represents the AAR. The AAR determined at each signal threshold is shown in red. At low thresholds non-specific signal noise results in bright pixels in non-ischemic territories causing an overestimation of the AAR. At higher thresholds the signal from the myocardial edema is masked by the presence of hemorrhage in the core of the infarct (arrow) and leads to an underestimation of the AAR.

The mean area of hemorrhage was 5.0% at the level of the infarct. There was a close correlation between hemorrhage and the MVO (r^2^ = 0.75, p < 0.01) and infarct volumes (r^2^ = 0.76, p < 0.01) (Figure 1). When ≥ 5% hemorrhage is present the AAR was underestimated by 50% at a 5 standard deviation threshold compared to a boundary detection tool (21.8% vs 44.0%, p < 0.05) (Figure 2). Estimation of myocardial salvage at 3 sd and 5 sd signal thresholds becomes unreliable in hemorrhagic infarcts as the apparent AAR becomes smaller than the actual infarct size.

## Discussion

Our findings demonstrate the feasibility of using T2* mapping to quantify myocardial hemorrhage following infarct reperfusion. Hemorrhage is frequently observed and is associated with large infarcts where MVO is present and is an indicator of poor myocardial salvage. Hemorrhage in the core of the infarct causes signal loss on T2-weighted imaging and boundary-detection is required to reliably assess the AAR.

## Conclusion

Studies using CMR to determine the AAR and myocardial salvage should use boundary detection methods for quantification as arbitrary signal thresholds are unreliable when hemorrhage is present. Post-reperfusion hemorrhage can be assessed with T2*-mapping and may provide an imaging marker of poor myocardial salvage.

